# The Development of Ebola Virus Outbreaks: A Review of Epidemiological Trends, Clinical Features, and Treatment Advances

**DOI:** 10.7759/cureus.74078

**Published:** 2024-11-20

**Authors:** Omar B Mohd, Seri Sawaqed, Mrinmoy Kundu, Reem A Ghannam, Ahmed B Mohd, Jehad F AlSamhori, Osama K Musallam, Abdalrahman Altiti, Hanan Hasan, Abdulrhman Khaity

**Affiliations:** 1 Faculty of Medicine, Hashemite University, Zarqa, JOR; 2 College of Medicine, Institute of Medical Sciences and SUM Hospital, Bhubaneswar, IND; 3 Faculty of Medicine, The University of Jordan, Amman, JOR; 4 Medical Laboratory, The Lab, Amman, JOR; 5 Faculty of Medicine, University of Khartoum, Khartoum, SDN

**Keywords:** ebola, ebola outbreak, ebola virus, global epidemiology, infectious disease outbreaks

## Abstract

The Ebola virus, a filovirus that causes human Ebola virus disease (EVD), has caused multiple epidemics in the African continent for about 50 years. Wild animals were the source from which the virus was transmitted to humans, and it spread among people through direct contact. The majority of Ebola outbreaks occurred in African nations, particularly in Sudan, the Democratic Republic of the Congo (DRC), Uganda, and Gabon. Although EVD is a lethal disease, it has posed a challenge to human efforts to comprehend its etiology, epidemiology, pathophysiology, pathogenesis, and the best methods for treatment and prevention. This review aims to present the history of Ebola epidemics in Africa, each subtype that caused an outbreak, the development of therapies and vaccines, and the significance of travel regulations.

## Introduction and background

The Ebola virus is an enveloped virus with a filamentous structure [1]. It also has a single-stranded, negative-sense RNA genome [2]. The genome encodes seven unique genes, and at least nine proteins can be expressed from these genes [1,3]. Generally, ebolaviruses belong to the Filoviridae family, specifically the Ebolavirus genus. There are five different types of ebolavirus: Taï Forest ebolavirus, Sudan ebolavirus, Reston ebolavirus, Bundibugyo ebolavirus, and Zaire ebolavirus [4].

The virus was first identified in 1976 during two simultaneous outbreaks in Sudan and the Democratic Republic of the Congo (DRC). Since then, there have been sporadic outbreaks of EVD in Africa, with the largest outbreak occurring between 2014 and 2016 [4,5]. On January 11, 2023, the Ministry of Health (MoH) of Uganda officially declared the conclusion of the Ebola disease outbreak caused by the Sudan ebolavirus. The outbreak had impacted nine districts within the country. Throughout the course of the outbreak, a total of 164 cases were reported, consisting of 142 confirmed cases and 22 probable cases [5].

The Ebola virus is transmitted through contact with the bodily fluids of infected individuals or animals [6]. After contact with the virus, symptoms might start between two and 21 days later, on average eight to 10 days. The symptoms of the Ebola virus include fever, headache, muscle pain, weakness, vomiting, diarrhea, and bleeding from various parts of the body [6,7]. Moreover, this virus causes a severe, frequently deadly disease called Ebola hemorrhagic fever (EHF) [8]. There is no specific treatment for the Ebola virus, and the mortality rate can be as high as 90% [9].

Although EVD is lethal, it has posed a challenge to human efforts to comprehend its etiology, epidemiology, pathophysiology, pathogenesis, and the best methods for treatment and prevention. This review aims to provide an overview of the current knowledge on Ebola virus outbreaks, including the history of Ebola epidemics in Africa, each subtype that caused an outbreak, the development of therapies and vaccines, and the significance of travel regulations. Overall, the study informs clinical practice, research priorities, and public health strategies for containing and mitigating the impact of Ebola virus outbreaks.

## Review

History of Ebola outbreaks

**Figure 1:**
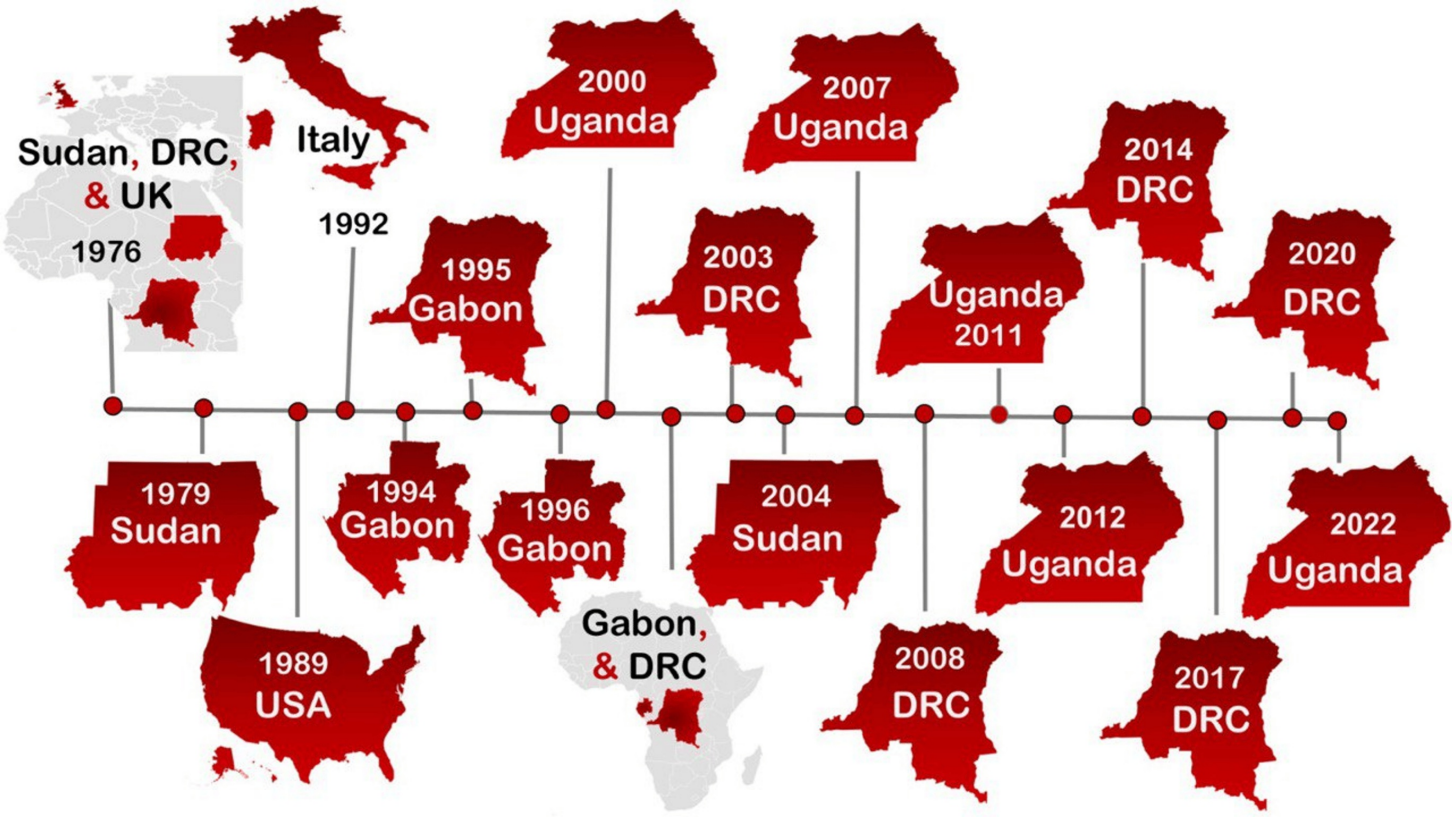
A timeline of Ebola virus outbreaks from 1976 to 2022, highlighting the location of each epidemic. DRC: Democratic Republic of the Congo This is an original figure created by the authors.

The majority of Ebola outbreaks occurred in African nations, particularly in Sudan, the DRC, Uganda, and Gabon. Figure 1 demonstrates the timeline of Ebola virus outbreaks. The public health concern of Ebola virus infection was highlighted by the several outbreaks that have been reported from non-endemic areas.

1976 in Sudan, the DRC, and the United Kingdom

In 1976, the first species of the Ebola virus was discovered near the Ebola River in the DRC, resulting in 318 reported cases, of which 280 resulted in fatalities, accounting for 88.1% mortality [10]. In addition, southern Sudan experienced a severe hemorrhagic fever outbreak around June 1976 and November 1976. In this outbreak, Ebola hemorrhagic fever was a unique clinical condition that had a massively high fatality rate (53%), and a lengthy recovery time for those who survived. Flu-like symptoms that initially comprised fever, headache, joint, and muscle pains were quickly followed by diarrhea, vomiting, chest pain, pain and dryness of the throat, and rash [10]. Bloody symptoms were common, appearing in 50% of recovered cases and almost 100% of mortalities [10]. Moreover, in one incident that was recorded in the United Kingdom, a contaminated needle accidentally inoculated the patient, resulting in a laboratory infection [11,12].

1979 in Sudan

In a rural southern Sudanese district, five families experienced 34 cases of the Sudan ebolavirus between July 31, 1979, and October 6, 1979. Direct physical contact with an infected person resulted in secondary transmission chains among the family units, accounting for 29 instances [4,13]. Emphasizing the significance of intimate contact in the spread of this illness, the risk of infection increased 5.1 times among those who were giving nursing care [13].

1994 in Gabon

Forty-nine individuals who suffered from hemorrhagic symptoms were admitted to the Makokou General Hospital in northern Gabon [6,14]. Following the initial polymerase chain reaction and blotting diagnosis of yellow fever virus in serum, a vaccination program was launched. A retrospective search for potential causative agents was conducted since some elements of this outbreak were unusual for yellow fever infection. It was discovered that the yellow fever virus and the Ebola virus co-existed in the pandemic. Ebola virus isolates from all three outbreaks had their glycoprotein (GP) and large (L) genomes partially sequenced, and the results revealed a base pair difference of 0.1%. All isolates were found to be very similar to the isolates of subtype Zaire ebolavirus from the DRC, according to sequencing results [15].

1996 in Gabon

The outbreak started during the first few days of February 1996. After skinning and chopping a chimpanzee corpse, they discovered that 18 individuals had experienced fever, headache, and bloody diarrhea [14]. The second outbreak of 1996 began in the fall of 1996 when the Zaire ebolavirus was isolated from two of six patients who were hospitalized. The pandemic, which had 60 cases and 45 fatalities, was reported to be over in March 1997 [16].

2000 in Uganda

The outbreak, which impacted the public and healthcare workers in Rwot-Obillo, was marked by fever and hemorrhagic symptoms [17-20]. The average attack rate for the Gulu district was 4.5 and 12.6/10000 cases when only contacts of laboratory-confirmed cases were considered, respectively. The secondary attack rate was 2.5% when approximately 5000 contacts were followed up for 21 days. The subtype isolated from this outbreak was the Sudan ebolavirus. Uganda publicly announced itself Ebola-free on February 27, 2001, 42 days after the last case was recorded [17-20].

2001 in Gabon and Congo

The first human cases in the area were linked to local hunting and animal interactions [17,21]. A total of 65 cases, with 53 fatalities (81.5% mortality rate), were reported in Gabon [17,21]. In Uganda, there were documented 425 cases, resulting in 224 deaths (52.7% mortality rate) [17,21].

2003 in Congo

One hundred thirty cases had epidemiological links, and 13 were confirmed in the lab [22]. Male patients made up 53 percent of the population. Ages ranged from five days to eighty. Direct contact with an infected person was necessary for transmission, particularly throughout families. Epidemiological data linked the initial three cases, which mostly involved hunters, to the entry of the Ebola virus (Zaire ebolavirus subtype) into the general population. In each of the three cases, interaction with nonhuman primates (gorillas) and other mammals (antelope) that had either been killed or found dead led to the development of the disease [6,14]. Subsequently, there were 35 documented cases, with 29 resulting in fatalities, indicating a mortality rate of 82.9% [16].

2004 in Sudan

The high suspicion of the disease rises after a total of seven people, five family members, and two healthcare workers developed signs and symptoms highly indicative of EHF. The prompt detection of the outbreak and the good strategy for detecting new cases with successive laboratory affirmations have led to a lesser progression of the outbreak, adding only 10 new cases to the outbreak [23].

2007 in Uganda

Uganda experienced its first EHF outbreak from August to December 2007 due to the Bundibugyo ebolavirus. Around 131 EHF cases were discovered during the outbreak response and assessment (44 suspected, 31 probable, and 56 confirmed) [24,25]. Between the initial EHF cases and the eventual detection of the Ebola virus and epidemic response, a considerable amount of time, approximately three months, elapsed, allowing for sustained person-to-person transmission of the virus [26].

2011 in Uganda

According to the government in Uganda, a patient's mortality in the Luwero district around May 6, 2011, was thought to be caused by a Sudan ebolavirus species [17,27]. It was discovered and identified by the research center in Uganda, which constructed the U.S. Centers for Disease Control and Prevention (CDC) Viral Hemorrhagic Fever Laboratory, which successfully recognized the Ebola virus from a blood sample [17,27].

2012 in Uganda

Three different outbreaks happened in Uganda in 2012 with the reappearance of the Sudan and Marburg viruses. The first outbreak happened in Kibaale in July with 11 confirmed cases just to end in August, to be followed by a second outbreak that also involved Ibanda in October with 15 more cases. In Nakasongola, Jinja, and Luwero districts, a third outbreak happened in November, with six cases being reported [14,28].

2014 in Congo

The seventh outbreak in Congo only affected the western province of Équateur, although it spread to several villages close to the town of Boende. Furthermore, since there was a strong relationship between the Ebola virus strain causing this outbreak and the strain causing the Kikwit outbreak in 1995, there is no link between this outbreak and the vast outbreak that was ongoing in West Africa at the same time [29,30]. Adding to that, it is thought that the first infected patient was a pregnant woman who had slaughtered a monkey; the virus spread then to the healthcare workers who had done a postmortem cesarean section on her. The total number of cases was 69, with 49 deaths (71.0%) [29,30].

2014-2016 in Guinea, Liberia, and Sierra Leone

The outbreak that occurred in Guinea, Liberia, and Sierra Leone in 2015 affected around 28,610 individuals and resulted in the death of 11,308 people, yielding a case fatality rate (CFR) of roughly 40%. The prediction by the CDC highlights that during the 2014-2016 West African EVD outbreak, there was a significant possibility of infection spreading to over 1.4 million individuals in Liberia and Sierra Leone combined [17].

2017 in Congo

International public health groups were notified of possible EVD cases in Likati, Bas Uélé, by the DRC's governments. In the initial report, there were eight confirmed cases, including two fatalities. A third fatality was recorded on May 12. Additionally, during the Institute National de Recherche Biomédicale testing in Kinshasa, two samples tested positive for Ebola Zaire [31,32].

2020 in the Democratic Republic of Congo

This outbreak, the 11th to occur in the DRC, began as the 10th was still actively spreading in eastern DRC [17]. The bulk of patients in this outbreak were most likely brought on by a recent spillover event or the recent introduction of the virus from an animal reservoir into the community, which was then spread from person to person, according to laboratory data [17]. Sequencing studies led to the identification of a few cases that appeared to be linked to the 2018 Équateur Province epidemic; these cases were most likely transmitted sexually or by a survivor relapsing [17].

2022 in Uganda

On September 20, 2022, Ugandan health officials declared an Ebola epidemic brought on by the Sudan virus following test confirmation of a case from a settlement in the Madudu sub-county [33]. A total of 23 deaths, including five among confirmed patients (CFR among confirmed cases: 28%), have been recorded from the districts of Mubende, Kyegegwa, and Kassandra as of September 25, 2022 [33,34]. There were 36 instances in total, with half of them being confirmed and the other third being under investigation. Since 2012, only one Ebola virus outbreak caused by the Sudan virus has occurred in Uganda [34].

Ebola virus disease complications

**Figure 2:**
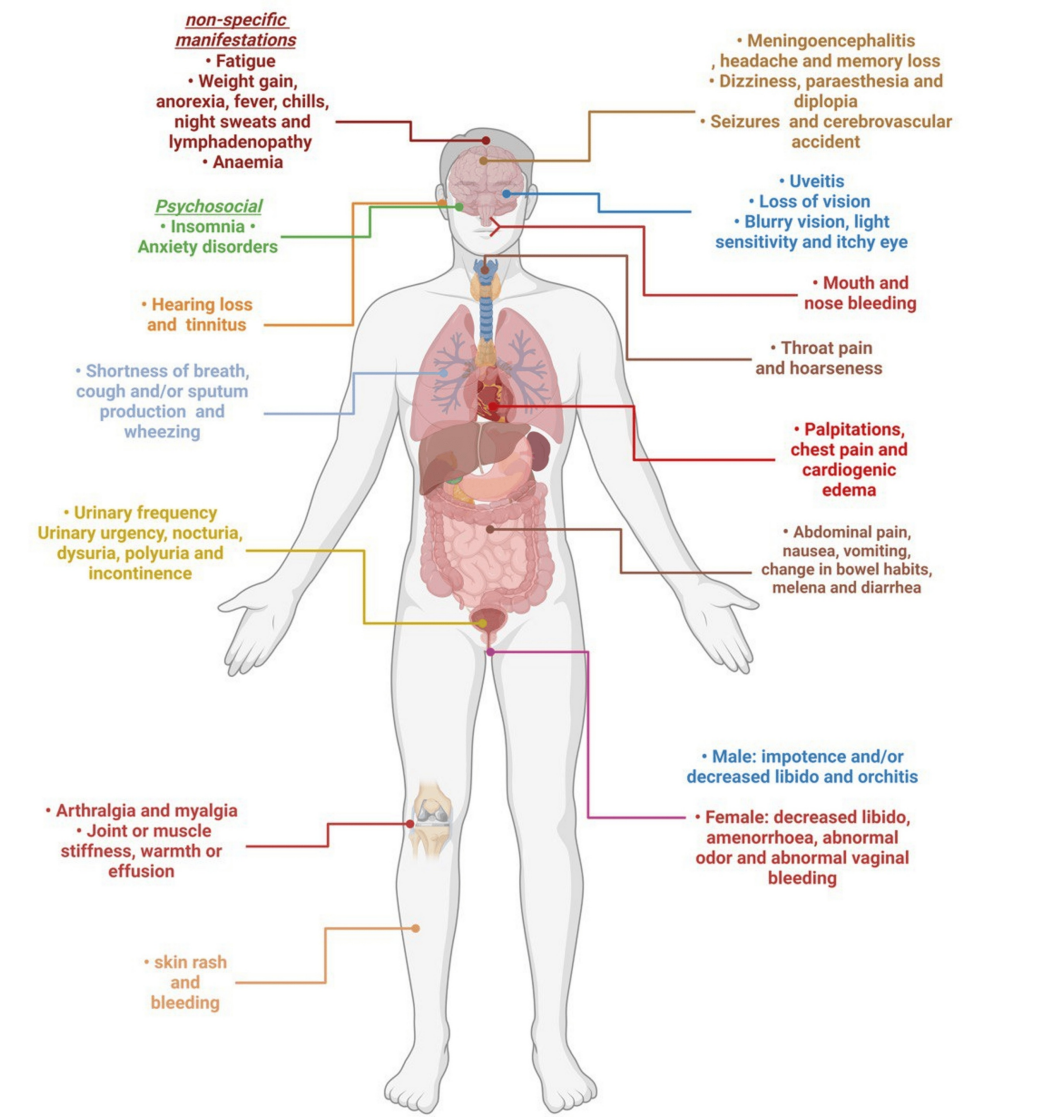
Demonstrates the complications and long-term effects of Ebola virus. This is an original figure created by the authors.

EVD is a severe and often fatal illness caused by the Ebola virus. One of the most common complications of EVD is hemorrhagic fever. This occurs when the virus damages blood vessels and causes bleeding throughout the body. Hemorrhagic fever can lead to shock, organ failure, and death. Another complication of EVD is multi-organ failure. The virus can damage multiple organs in the body, including the liver, kidneys, and lungs. This can lead to respiratory failure, kidney failure, and other serious health problems [27,35]. Furthermore, unspecific symptoms such as fatigue or loss of taste, an inflammation that can result in contingent damage to the eye causing blindness like uveitis, and migratory arthralgia constitute a common fraction of all affected survivors. Moreover, it was theorized that the viral load of the disease could have a role in developing uveitis or any other ocular symptoms [27,35]. Figure 2 demonstrates the complications and long-term effects of the Ebola virus.

Neurological complications are also possible with EVD. The virus can cause inflammation in the brain and spinal cord, leading to symptoms such as confusion, seizures, and paralysis. In addition to these physical complications, EVD can also have psychological effects on survivors. Many survivors experience post-traumatic stress disorder (PTSD) or depression due to their experience with the disease [27,35].

Treatment and vaccinations

There is currently no specific treatment or vaccine for EVD, and management of the disease is primarily supportive. Treatment focuses on managing symptoms such as fever, vomiting, diarrhea, and dehydration. Patients are also given fluids and electrolytes to maintain their fluid balance [36-38].

In addition to supportive care, several experimental treatments have been developed for EVD. These treatments include monoclonal antibodies, antiviral drugs, and convalescent plasma therapy. Monoclonal antibodies are laboratory-made proteins that target specific parts of the Ebola virus. Inmazeb (Odesivimab, Maftivimab, and Atoltivimab), a combination of three monoclonal antibodies, was the first therapy for Zaire ebolavirus infection to be licensed by the FDA in 2018. Additionally, in 2020, it authorized the use of Ansuvimab, the human monoclonal antibody Ebanga [39-41]. Antiviral drugs work by inhibiting the replication of the virus in the body. Convalescent plasma therapy involves giving patients blood plasma from individuals who have recovered from EVD. A randomized controlled study was performed during the Ebola outbreak in the DRC in 2018-2020 to assess these therapies and two others. When it came to lowering EVD mortality, Inmazeb and Ebanga were both superior to the other medications [39].

As a consequence of the severe EVD pandemic in Africa from 2013 to 2016, many vaccines have demonstrated protection in human and nonhuman primates. There are now two approved and in-use vaccines: a two-dose combination of Zabdeno (Ad26.ZEBOV) and Mvabea (MVA-BN-Filo) and Ervebo (rVSV-ZEBOV-GP) [39,42]. The Global Advisory Committee on Vaccine Safety stated the overall safety profiles of both the rVSV-ZEBOV-GP and the Ad26.ZEBOV vaccines. In addition, ZEBOV/MVA-BN-Filo vaccine designs are reassuring, and both vaccines represent medical advancements against EVD [43].

**Figure 3:**
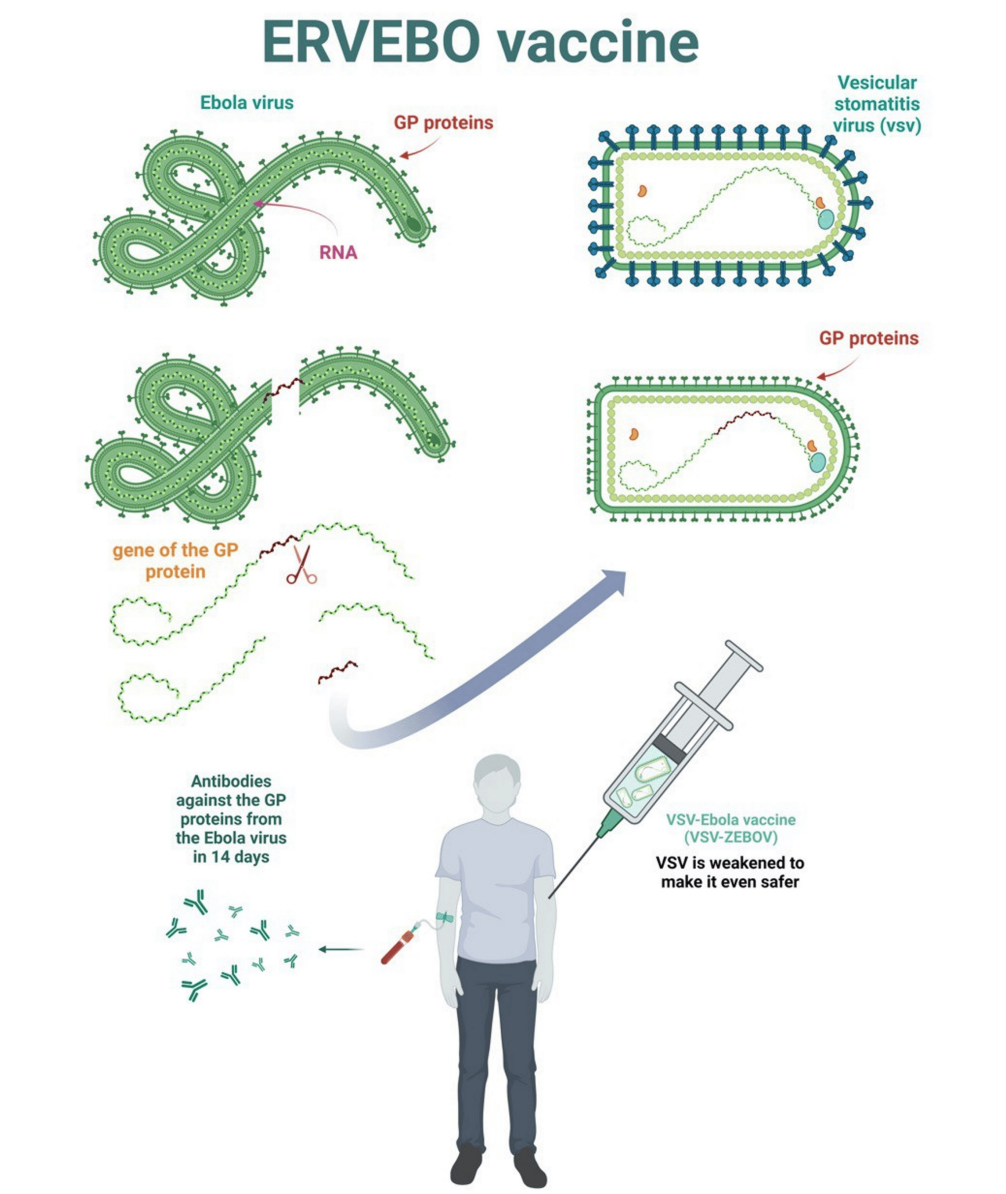
Demonstrates the mechanism of the VSV-Ebola vaccine. VSV: vesicular stomatitis virus; GP: glycoprotein This is an original figure created by the authors.

Ebola virus attacks cells by attaching to them via glycoprotein (GP) covering the virus. It then enters the cells and forces them to produce more viruses. To be protected against the Ebola virus, the body must produce antibodies to neutralize the GP of the virus [44,45]. This process requires the body to be in contact with the virus but without the risk of getting the disease. And this is the role of the vesicular stomatitis virus-Ebola vaccine. The GP is brought into the bloodstream but through the vesicular stomatitis virus (VSV), which can stimulate the immune system without becoming life-threatening, causing symptoms no worse than those of the flu [42]. To make the vaccine, researchers took the gene responsible for GP from the Ebola virus and inserted it into the VSV, thus the new virus has the GP of the Ebola virus on its surface. They also weakened the virus to make it even safer for humans [46]. Figure 3 demonstrates the mechanism of the VSV-Ebola vaccine.

The FDA has authorized Ervebo as a single-dose administration to prevent infection caused by the Zaire ebolavirus in people older than 18 years. Clinical studies have shown that a single vaccine dosage induces a rapid antibody response in 14 days. Furthermore, a cluster-randomized trial conducted in Guinea assessed the drug's efficacy. In this trial, 3,775 participants who had close contact with diagnosed EVD patients and their contacts were vaccinated immediately. No one who was immunized developed EVD 10 days or more after immunization. According to a study, the Ervebo vaccine has high protective efficacy and effectiveness in preventing EVD [47].

Juan-Giner and colleagues researched to assess the safety of the Ervebo vaccine and documented some adverse effects following the vaccination. Approximately 75% of participants reported adverse effects such as myalgia, fever, arthralgia, fatigue, and headache. Most adverse effects were recorded three days after the vaccination and typically resolved within three to four days [46].

The Zabdeno/Mvabea vaccination is given in two doses: the first dosage is Zabdeno, and the second dose of Mvabea (MVA-BN-Filo) is given around two months later. Because urgent protection is required during an epidemic, this preventative two-dose regimen is not appropriate. Regarding efficacy, preclinical research shows that nonhuman primates were completely protected against the Ebola virus by a Zabdeno/Mvabea prime-boost vaccine [48]. Zdeno/Mvabea is safe and induces both CD8+ and CD4+ T cell responses in vaccination recipients and potent antibody responses. At an eight-week dosing interval, Zabdeno/Mvabea showed the most remarkable protective efficacy in nonhuman primates. Shorter dosage intervals offered less protection. It is worth mentioning that Zabdeno cannot multiply in humans, in contrast to Ervebo [48].

Recommendations

Numerous challenges have faced the healthcare system while controlling the Ebola virus in West African countries. These include entrenched poverty and food insecurity, civil conflicts emerging in these countries, endemic multi-morbidities, poor public health infrastructure, and limited access to modern health care [49].

There are many recommendations from the CDC and the WHO for the general population to avoid infection with Ebola. These include avoiding touching ill people's blood and bodily fluids such as vaginal secretions, semen, amniotic fluid, breast milk, vomit, perspiration, saliva, feces, and urine. Furthermore, avoid touching the body of a person who died from the Ebola virus or is suspected of having the virus during a funeral or burial procedure. In addition, reducing the possibility of wildlife to human transmission by reducing the interaction with nonhuman primates that are diseased, as well as by avoiding eating their raw flesh [50].

The fight against Ebola viruses involves various regulations, restrictions, and proactive measures. Geographical factors, such as dense forests serving as natural reservoirs, pose challenges to containment efforts. Additionally, limited infrastructure and difficult terrain hinder healthcare services and supply delivery. Economic considerations are crucial, as outbreaks impact sectors like tourism and trade. International organizations like WHO and CDC must establish regulations, surveillance systems, and resource-sharing protocols. Travel restrictions and screening measures help limit virus spread across borders. Rapid diagnostic tests offer quick results in resource-limited settings, aiding early detection and response. Strengthening healthcare infrastructure is vital, ensuring adequate facilities and trained personnel. Ongoing research focuses on improving treatments, vaccines, and diagnostics. Cultural practices, like safe burial campaigns, are important for community engagement. Overall, addressing these factors enhances the global response to Ebola outbreaks, minimizing their impact on affected communities [51].

## Conclusions

Firstly, it is important to note that Ebola outbreaks are likely to continue occurring in the future. The virus is endemic in certain regions of Africa, and there is a risk of new outbreaks emerging as a result of human-animal interactions or other factors. However, there are also reasons to be optimistic about our ability to contain and manage Ebola outbreaks in the future. Advances in medical treatments and vaccines have already made a significant impact on reducing mortality rates from Ebola.

In addition, improvements in surveillance and response systems have helped to detect and respond to outbreaks more quickly. This includes early warning systems that use data from social media and other sources to identify potential outbreaks before they become widespread. Looking further ahead, there is also potential for new technologies such as gene editing and artificial intelligence to play a role in preventing or treating Ebola outbreaks.
